# Synovial immune control failure in osteoarthritis: from maintenance of tissue homeostasis to inflammatory niche formation

**DOI:** 10.3389/fimmu.2026.1889424

**Published:** 2026-08-31

**Authors:** Kangyi Hu, Yongjia Song, Jiaxing Pan, Zhiqiang Wang, Weiran Li, Chaoqun Yan, Yongpeng Xue, Xiao Jia, Luhao Yu, Min Song, Jiamin Bao

**Affiliations:** 1 Clinical College of Traditional Chinese Medicine, Gansu University of Chinese Medicine, Lanzhou, China; 2 School of Rehabilitation Science, Shanghai University of Traditional Chinese Medicine, Shanghai, China

**Keywords:** fibroblast-like synoviocytes, inflammatory niche, macrophages, osteoarthritis, synovial immune control failure, synovium

## Abstract

Osteoarthritis (OA) is a whole-joint disease involving cartilage, synovium, subchondral bone, and neurovascular structures. Synovitis, damage-associated molecular patterns, and interactions between macrophages and fibroblast-like synoviocytes have been widely studied. However, a tissue-level explanation is still lacking for how low-grade and fluctuating synovial responses become persistent inflammation. We therefore propose a framework of synovial immune control failure. In this framework, the synovium does more than participate in inflammation. By clearing intra-articular damage-related material, limiting the duration of inflammation, maintaining stromal homeostasis, and regulating the entry of peripheral immune cells, it helps determine whether local joint inflammation can return to a low-level state. When damage-related inputs persist and debris clearance, inflammatory resolution, and resetting of cellular states do not occur in parallel, macrophages, fibroblast-like synoviocytes, and the vascular-interstitial interface may form a mutually sustaining pathological cellular network. Disease-associated cellular states and their interactions may then be retained locally in the synovium, forming what this article defines as an inflammatory niche. This niche may form bidirectional feedback with cartilage degeneration and subchondral bone remodeling. However, the synovium is not the common initiating tissue in all patients with OA. This framework may be most relevant to disease subtypes with persistent or recurrent synovitis, myeloid cell activation, and abnormal stromal remodeling. We further discuss the evidence base, boundaries, testable predictions, and potential implications of this model for OA patient stratification and synovium-targeted intervention.

## 1 Introduction

Osteoarthritis (OA) is a whole-joint disease involving articular cartilage, synovium, subchondral bone, and neurovascular structures. Its main pathological features include cartilage matrix degradation, abnormal subchondral bone remodeling, and osteophyte formation (1, 2). However, structural degeneration alone cannot capture all clinical and biological features of OA (3, 4). Some patients have long-standing low-grade synovial inflammation. Pain severity does not always match structural damage on imaging, and patients differ markedly in disease progression and treatment response (5). These observations indicate that OA is not a simple process of mechanical wear. It is a heterogeneous disease shaped by tissue injury, local inflammation, and failed repair.

Synovitis is a common pathological feature of OA, but it varies greatly among individuals. Longitudinal imaging studies show that synovitis can appear before definite radiographic structural changes and is associated with later pain and structural progression (6, 7). Recent single-cell sequencing and spatial omics studies further show disease-associated changes in the composition and states of macrophages, fibroblast-like synoviocytes (FLS), and other immune and stromal cells in OA synovium (8, 9). These findings support a role for the synovium in maintaining OA inflammation and mediating tissue crosstalk. They do not prove that the synovium is the common initiating tissue in all patients with OA. The degree and duration of synovitis, and its relationship with cartilage and subchondral bone lesions, may differ among patients. Previous studies have described many pathological features of OA synovitis at the levels of inflammatory mediators, cell subsets, and signaling pathways. Yet the process by which low-grade, focal, and fluctuating synovial changes become persistent tissue inflammation remains poorly understood. Under physiological conditions, resident macrophages, FLS, and the vascular-interstitial interface in the synovium jointly participate in intra-articular debris clearance, maintenance of synovial-fluid and matrix environments, and regulation of immune-cell recruitment (10). These functions limit the intensity, duration, and spread of local inflammation. As mechanical loading, matrix injury, cell death, and metabolic stress continue to accumulate, synovial homeostatic functions may gradually become impaired. Low-grade inflammation may then shift from a transient injury response to a persistent pathological state.

**Figure 1:**
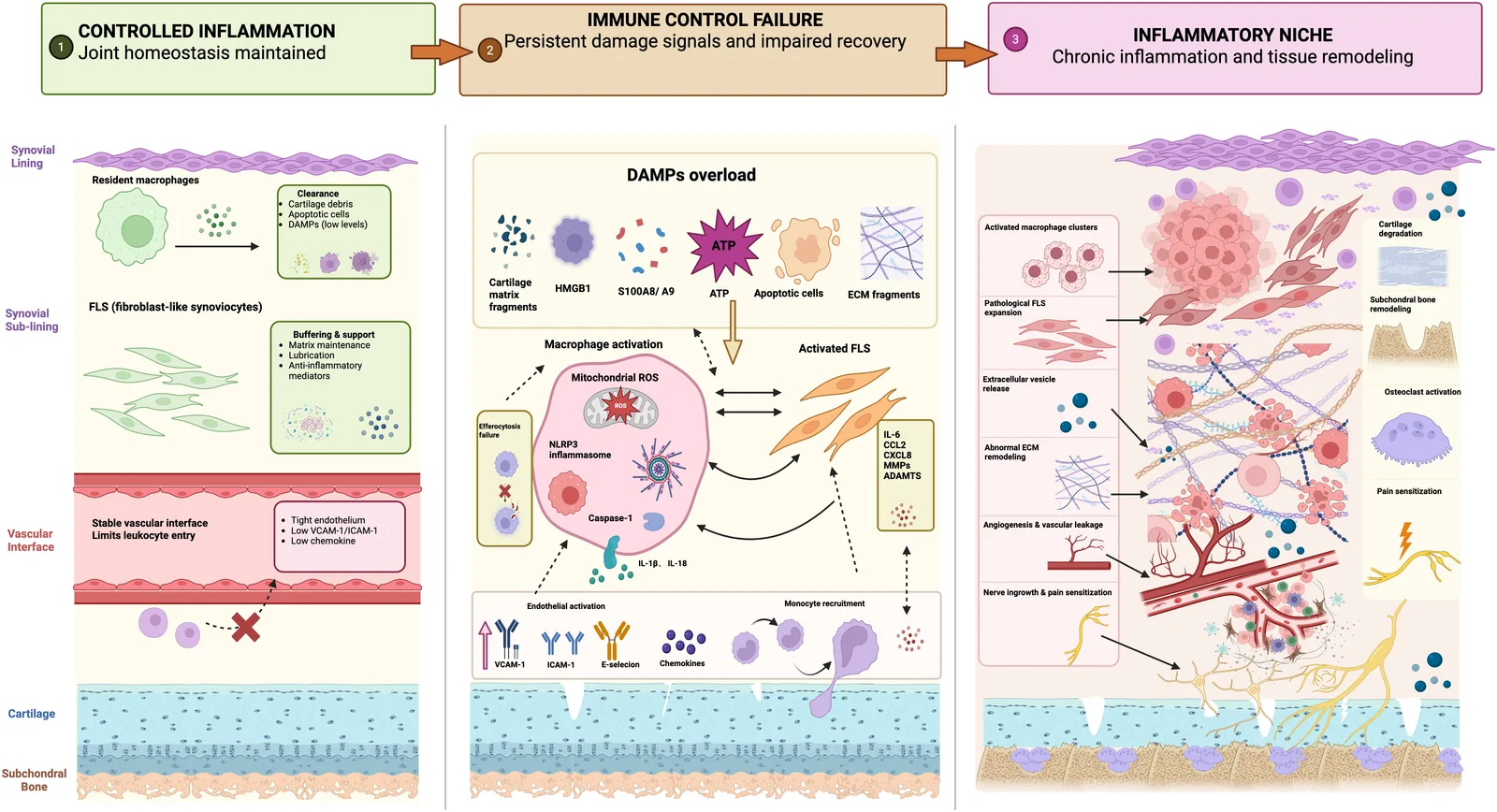
Proposed transition from a reversible synovial response to synovial immune control failure and inflammatory niche formation in OA. Under homeostatic conditions or during a reversible response to minor injury, resident synovial macrophages, fibroblast-like synoviocytes (FLS), and the vascular–interstitial interface cooperate to clear intra-articular debris, maintain the synovial-fluid and extracellular-matrix environment, and regulate the recruitment of circulating immune cells. When mechanical stress and tissue injury persist, continued generation of damage-related signals may coincide with impaired efferocytosis, delayed inflammatory resolution, altered macrophage and FLS states, endothelial activation, and prolonged myeloid-cell recruitment. Inflammasome activation and metabolic or mitochondrial stress may amplify inflammatory output under specific conditions. If these responses fail to resolve after the initiating damage input has diminished, disease-associated cellular states and their interactions may remain locally retained, forming what is proposed here as a synovial inflammatory niche. This niche may participate in reciprocal communication with cartilage and subchondral bone and thereby prolong synovial inflammation and tissue remodeling. The model does not imply that the synovium is the universal initiating tissue in osteoarthritis. The schematic is based on representative studies of resident synovial macrophages, macrophage clearance, FLS states, macrophage–FLS communication, and experimental modulation of synovial inflammation. Created with BioRender.com.

Based on these observations, this article proposes synovial immune control failure as a tissue-level framework for pathological transition. Under physiological or early injury conditions, resident macrophages, FLS, and the vascular-interstitial interface clear intra-articular damage-related material, maintain the local matrix environment, and restrict the spread of inflammation. In this way, low-level immune responses remain within a regulatable range. With persistent mechanical stress, tissue injury, and metabolic abnormalities, the ability of the synovium to restore homeostasis may gradually weaken. Macrophage clearance function, FLS states, and vascular-interstitial regulatory networks then change, promoting the transition from a transient and localized inflammatory response to persistent inflammation. During this process, abnormal cellular states and their interactions may become progressively stabilized in local synovial tissue and may form a pathological microenvironment that maintains inflammation and tissue remodeling. This microenvironment is referred to here as the synovial inflammatory niche. The following sections discuss this framework from the perspectives of the tissue basis of synovial immune homeostasis, the pathological process of immune control failure, inflammatory niche formation, and crosstalk with other joint tissues (**Figure 1**).

## 2 Tissue basis of synovial immune homeostasis

The synovium consists of a lining layer facing the joint cavity and a sublining layer rich in vessels, stroma, and immune cells. Because it lacks a continuous basement membrane, the synovium is not a closed epithelial covering. It is an open interface connecting synovial fluid, periarticular tissues, and the peripheral circulation (11, 12). Cellular debris, matrix wear products, and metabolites generated during joint motion can directly contact the synovial lining and exchange with deeper stroma through interstitial fluid and vascular networks. Synovial homeostasis therefore depends not only on which cells are present, but also on where they are located and how they relate to one another in different spatial regions. Single-cell sequencing studies show that macrophages, fibroblast-like synoviocytes (FLS), vascular endothelial cells, pericytes, and other immune cells in the synovium have clear regional distribution patterns (8, 9). Cells in the lining layer directly process signals from the joint cavity, whereas the sublining layer supports stromal maintenance, material exchange, and cell migration. This spatial division provides a basis for understanding how the synovium maintains a relatively stable local environment during continuous mechanical loading and minor tissue wear.

### 2.1 The synovial lining interface: cooperation between resident macrophages and lining FLS

The synovial lining layer is mainly composed of macrophage-like synoviocytes and FLS (13). Both cell types face synovial fluid directly, but they have different functions. Resident macrophages clear apoptotic cells, cartilage matrix fragments, and other intra-articular debris. Lining FLS synthesize lubricin, hyaluronic acid, and related matrix components, thereby maintaining synovial-fluid properties and a low-friction environment on the joint surface (14–17). This spatial proximity allows clearance of damage-related material and maintenance of the synovial-fluid environment to continue at the same tissue interface.

The role of resident macrophages in the synovial lining is not limited to phagocytosis. In mouse joints, Culemann et al. found that CX3CR1+ resident synovial macrophages form a barrier-like structure along the lining layer and restrict the entry of peripheral inflammatory cells into the joint cavity during experimental arthritis (10). That study mainly used inflammatory arthritis models and does not directly prove that the same barrier mechanism exists in OA synovium. Even so, it suggests that tissue-resident macrophages may help maintain the joint boundary through stable spatial positioning and cell-cell connections.

Single-cell studies of human OA synovium have identified different macrophage states with features related to tissue residence, phagocytic clearance, lipid handling, and immune regulation. Together with studies of healthy synovium and inflammatory joint disease, these findings show that molecules such as MERTK, CD163, FOLR2, and TREM2 can be present in some tissue-resident or immunoregulatory macrophage populations (18–20). These markers do not define a fixed category of protective macrophages, because their expression is affected by sampling site, disease stage, and the local environment. Current findings more appropriately indicate that OA synovial macrophages show clear state heterogeneity, and that some cells retain transcriptional features related to tissue maintenance and debris handling. Whether these cells gradually decrease or undergo functional changes during OA progression still lacks longitudinal observation and lineage-tracing evidence.

Lining FLS also have clear regional features. FLS with high PRG4 and HAS1 expression are mainly distributed in the lining layer and are associated with lubricin production, hyaluronic acid synthesis, and synovial-fluid homeostasis (21, 22). Synovial fibroblast atlas studies further show that PRG4-high lining FLS and THY1-high sublining FLS represent two major spatial and functional states, and that their molecular features can change with the tissue environment (23, 24). It is reasonable to propose that lining FLS influence the distribution of injury products and cell contacts in the joint cavity by maintaining synovial fluid and the surface matrix environment. However, defining them directly as an independent immune barrier that blocks DAMP diffusion still requires more direct functional evidence.

Thus, homeostasis at the synovial lining interface cannot be attributed to a single cell type. Resident macrophages mainly handle debris and maintain the tissue boundary, whereas lining FLS maintain synovial fluid and the surface matrix environment. Their spatial cooperation allows low-level injury inputs generated during joint motion to be processed promptly near the joint cavity.

### 2.2 The sublining layer: regulatory roles of stromal tissue and the vascular interface

The sublining layer lies deep to the lining layer. It contains blood vessels, lymphatic vessels, FLS, macrophages, and other immune cells, and it is supported by extracellular matrix (25, 26). Unlike the lining layer, which directly contacts synovial fluid, the sublining layer mainly provides tissue support, nutrient exchange, and cell migration, while connecting the local joint environment with the peripheral circulation.

Sublining FLS have strong capacities for matrix production and maintenance of tissue structure. Single-cell studies often associate them with molecular features such as THY1/CD90, PDPN, COL1A1, and CXCL12 (16, 27, 28). These cells are mainly distributed around vessels and in deep stroma. By synthesizing and renewing extracellular matrix, they provide spatial support for vessels, immune cells, and other tissue components. Some sublining FLS also express chemokines involved in cell positioning and local communication, but their physiological role should not be equated simply with inflammatory cell recruitment. Under homeostatic conditions, these signals also help maintain normal cell distribution and intercellular connections within the tissue.

The synovial lining layer itself is avascular, so tissue nutrition, oxygen supply, and metabolic waste clearance depend mainly on microvessels in the sublining layer. Endothelial cells, pericytes, and perivascular FLS maintain vascular structure and regulate permeability, leukocyte adhesion, and cell extravasation (29, 30). Entry of circulating immune cells into the synovium is not completely blocked. It is regulated by local signals and vascular state. During low-level tissue injury, limited cell migration may contribute to clearance and repair. When the stimulus weakens, cell recruitment should also decline. The homeostatic function of the vascular-interstitial interface therefore lies mainly in coordinating local tissue needs with peripheral cell input, rather than forming an absolutely closed barrier.

In addition to macrophages, the sublining layer can contain T cells, B cells, mast cells, dendritic cells, and other immune cells (31). Their number and composition differ markedly among individuals and joint regions. Current evidence does not establish a universal dominant role for any adaptive immune cell type in OA synovial homeostasis. However, these cells can communicate locally with FLS, macrophages, and perivascular cells and may influence how the tissue responds to injury.

**Figure 2:**
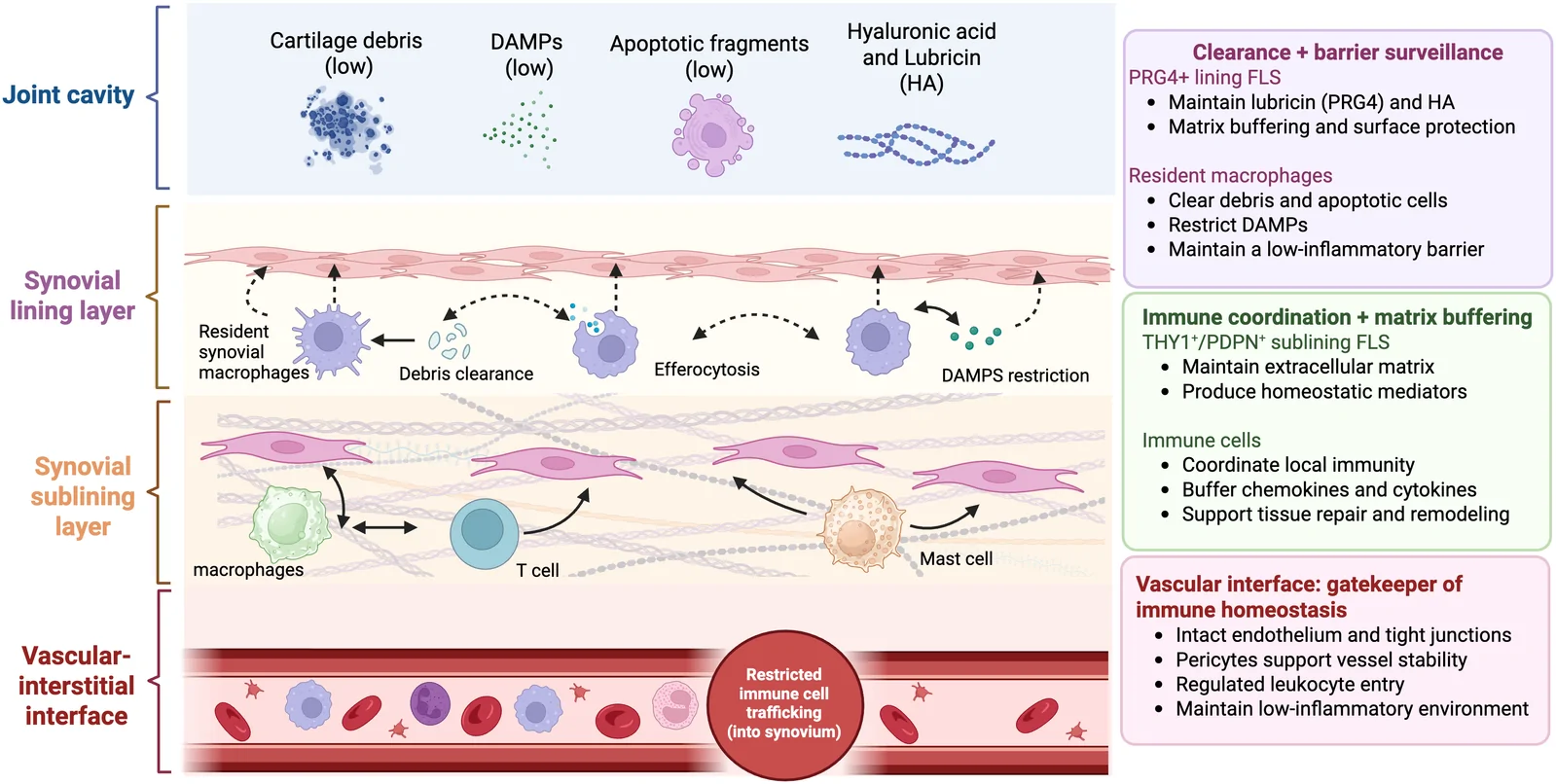
Cellular architecture of the synovial immune control system. This schematic shows the spatial organization of the synovial immune control system. The synovium is organized into the joint cavity/synovial fluid compartment, the synovial lining layer, the synovial sublining layer, and the vascular-interstitial interface. In the joint cavity, low levels of cartilage debris, DAMPs, apoptotic fragments, hyaluronic acid, and lubricin are present under homeostatic conditions. In the lining layer, PRG4^+^ lining FLS and resident synovial macrophages form a functional barrier-like interface. PRG4^+^ FLS maintain lubricin and hyaluronic acid production, support matrix buffering, and protect the synovial surface, whereas resident macrophages clear debris and apoptotic cells, restrict DAMP accumulation, and maintain a low-inflammatory barrier. In the sublining layer, THY1^+^/PDPN^+^ sublining FLS, macrophages, T cells, and mast cells are embedded within the extracellular matrix and participate in immune coordination, chemokine buffering, and tissue remodeling potential. The vascular-interstitial interface, composed of endothelial cells, pericytes, and circulating immune cells, acts as a gatekeeper that regulates leukocyte entry and preserves immune homeostasis. Together, these spatially organized cellular compartments maintain a low-inflammatory joint microenvironment through clearance, buffering, immune coordination, and restricted immune cell trafficking. Created with BioRender.com.

Overall, the synovial lining and sublining layers are not independent regions. The lining interface contacts and processes signals from the joint cavity, while the sublining layer maintains stromal structure and regulates exchange between local tissue and the peripheral circulation. Resident macrophages, spatially distinct FLS states, and vascular-associated cells thereby form a continuous tissue system. This system allows the joint to return to a relatively stable local state despite repeated low-level injury. The next section discusses which changes in these homeostatic functions may be sufficient to transform transient synovial responses into persistent inflammation (**Figure 2**).

## 3 Synovial immune control failure: from impaired homeostatic recovery to persistent inflammation

DAMP-mediated innate immune activation, synovial macrophage heterogeneity, pathological FLS phenotypes, and their interactions have become major topics in current osteoimmunology research on OA. These studies mainly explain which cells and molecules participate in synovial inflammation. They less often distinguish a transient injury response from persistent inflammation that cannot terminate spontaneously. Increased inflammatory mediators, more immune cells, or synovial thickening can reflect the presence and degree of a local response. They do not show whether the synovium can restore homeostasis after the stimulus weakens.

Compared with existing osteoimmunological frameworks, the synovial immune control failure model does not add a new inflammatory cell type or signaling pathway. Instead, it includes the reversibility of inflammation in pathological assessment. Similar degrees of synovial activation may have different outcomes. Some responses resolve as injury decreases, whereas others persist after the original stimulus has weakened. This article defines the latter condition as synovial immune control failure. In this state, after injury input declines, the synovium still cannot return promptly to a relatively stable low-inflammatory state. Its manifestations may include insufficient clearance of damage-related material, delayed inflammatory resolution, weakened homeostatic functions of resident cells, persistence of pathological cellular states, or prolonged abnormal immune-cell recruitment and retention. These changes do not have to occur together, and they do not form a fixed linear cascade. From this perspective, persistent synovitis cannot be judged only by whether a given inflammatory pathway is activated. The more important questions are whether the local response remains synchronized with the current injury burden and whether synovial cells can re-establish homeostatic programs after the stimulus weakens.

### 3.1 From reversible synovial responses to impaired homeostatic recovery

Synovitis does not necessarily indicate immune control failure. Imaging and histological studies show that synovitis can appear before definite radiographic OA and is associated with pain and later structural progression (3, 6). At the same time, OA synovitis differs greatly in severity, anatomical distribution, and duration. Some patients show only low-grade or focal changes, and synovial thickening and effusion can fluctuate with joint load and observation time (32, 33). These phenomena indicate that early or mild synovial responses may still be reversible.

Joint motion and minor tissue injury continuously generate small amounts of cellular debris, matrix degradation products, and metabolic signals. Transient immune activation helps process injury-related material and support tissue repair. Its pathological significance depends on whether the response can terminate in time. A single imaging examination or measurement of inflammatory mediators cannot distinguish a transient response from impaired homeostatic recovery. Even if a cellular state is associated with pain or structural damage, this does not confirm that it participates in inflammation persistence.

The protective role of resident macrophages further shows that synovial immune activity should not be equated simply with pathogenic inflammation. As noted above, resident macrophages in the lining layer can maintain tissue boundaries and restrict inflammatory spread (10). Nonselective depletion of intra-articular macrophages may not improve disease and can instead aggravate acute synovitis in injury models (34). Therefore, the key change in OA synovium may not be an overall increase in macrophage number. It may be whether tissue-resident, clearance-related, and repair-related functions are preserved.

Immune control failure has a clear temporal dimension. Only when inflammatory mediators, cell recruitment, or abnormal cellular states persist after injury stimuli have weakened can we infer that the ability of the synovium to restore homeostasis may be impaired. Most current OA studies are based on cross-sectional human tissues or animal samples collected at fixed time points. Direct evidence for this dynamic process remains limited.

### 3.2 Persistence of damage-related signals: increased generation, insufficient clearance, and delayed resolution

Damage-related signals in OA joints can arise from cartilage matrix degradation, subchondral bone microinjury, cell death, crystal deposition, and metabolic stress. HMGB1, S100A8/A9, ATP, and some extracellular matrix degradation fragments are representative DAMPs that have been studied extensively (35). Different DAMPs have different strengths of evidence and modes of action in OA. *In vitro* studies show that combined stimulation with HMGB1 and LPS can enhance inflammatory, migratory, and matrix-degrading phenotypes in human OA-FLS (36). In experimental OA, S100A8/A9 can promote recruitment of Ly6Chigh monocytes to the synovium and is associated with aggravated synovitis and joint structural damage (37). Hyaluronic acid degradation fragments can also affect monocyte, macrophage, and fibroblast responses, but their biological effects depend on fragment size, source, and experimental conditions (38–40). These findings suggest that DAMPs can help translate tissue injury into local immune responses. They should not be treated as a uniform class of molecules that inevitably causes persistent inflammation.

The residence time of damage-related material also depends on synovial clearance capacity. In advanced human knee OA synovium, apoptotic cell burden is increased and macrophage efferocytosis is impaired. OA synovial fluid can also inhibit clearance of apoptotic cells by peripheral-blood-derived macrophages (41). Animal models of post-traumatic OA similarly show accumulation of apoptotic cells in the synovium and impaired efferocytosis (42). In addition, reduced GAS6-related signaling and altered CD11b lactylation under hyperglycemic conditions may affect macrophage recognition and engulfment of apoptotic cells and are associated with aggravated experimental OA (43, 44).

Persistent exposure to DAMPs in OA joints may result from continuous release of damage-related signals by cartilage, subchondral bone, and other joint tissues. It may also be related to reduced ability of synovial macrophages to clear apoptotic cells and tissue debris. Apoptotic cells that are not processed in time can undergo secondary necrosis and release more intracellular components, further prolonging local exposure to danger signals. However, current evidence is not sufficient to define impaired efferocytosis as the common initiating mechanism of all OA synovitis. Related studies are mainly concentrated in post-traumatic OA, models associated with metabolic abnormalities, and relatively late-stage human OA samples. Impaired clearance is therefore more likely to be a specific manifestation of blocked synovial homeostatic recovery in some OA phenotypes.

Persistently retained ATP, crystals, cellular debris, and oxidative stress signals can also amplify local responses through inflammasomes. NLRP3 inflammasome assembly can be detected in OA synovium, promoting caspase-1 activation and mature IL-1beta and IL-18 release (45). Its expression is associated with macrophage infiltration and inflammatory severity (46). In some experimental OA models, targeting macrophagic PIM-1 reduces synovitis and joint pathology by inhibiting NLRP3 activation related to mitochondrial ROS/Cl- efflux (47). These intervention studies support the involvement of NLRP3 in inflammatory amplification under specific conditions (48, 49), but they are not sufficient to define it as the unified core of synovial immune control failure. Its role is closer to converting persistent injury signals into inflammatory output.

Whether inflammation persists also depends on whether resolution programs can start normally after the stimulus weakens. Under physiological conditions, inflammatory mediators are gradually cleared, immune-cell recruitment declines, apoptotic cells are processed, and tissue repair programs are re-established (35, 50). If these processes are blocked, the synovium may maintain high inflammatory output even when new injury input has decreased. Persistent DAMP exposure and NLRP3 activation are therefore more likely to prolong or amplify early inflammation. Whether the synovium shifts further into a persistent state depends on whether macrophages, FLS, and other local cells can exit inflammatory and tissue-remodeling programs.

### 3.3 Persistence of cellular states: macrophages, FLS, and adaptive immune regulation

Single-cell studies show that macrophages and FLS in OA synovium are not homogeneous populations, and that different cellular states vary among patients and sampling regions (8, 9). These studies provide a basis for identifying disease-associated cellular states. However, most samples were collected at a single time point, and many came from patients undergoing late-stage joint replacement. Thus, current evidence can confirm marked cellular heterogeneity in OA synovium, but longitudinal observations are still lacking to determine whether these states are transient injury responses or long-term retained states during disease progression.

Differences among synovial macrophages involve cell origin, spatial location, and functional state. Cells with features of tissue residence, phagocytic clearance, and lipid handling can coexist with states related to chemokine secretion, inflammatory sensing, and tissue remodeling (20, 51, 52). Redistribution of macrophage subsets and enhanced M-CSF-related signaling have been observed in post-traumatic OA synovium (51). Human OA synovium studies also show disease-associated communication between monocyte-derived macrophages and FLS (53). These changes cannot be summarized by the M1/M2 binary framework. Traditional classification does not reflect differences between tissue-resident cells and peripherally recruited cells, and it overlooks the continuous changes in macrophage states driven by the local environment. The key issue in OA synovium may therefore be not only an increase in a certain macrophage type, but also whether pre-existing clearance and tissue-maintenance states are preserved, and whether recruited cells can exit inflammatory programs after injury weakens.

Metabolic changes provide another clue for understanding the plasticity of macrophage states. In LPS-stimulated macrophages, songorine reduces the extracellular acidification rate and the expression of glycolysis-related molecules such as HK2, PFKFB3, and LDHA. It also improves oxidative phosphorylation, ATP production, and mitochondrial function. In surgically induced OA rats, this intervention is accompanied by reduced synovitis and cartilage damage (54). After AICAR activates SIK1, it can also regulate glucose and lipid metabolism in macrophages, reduce inflammatory mediator expression, and alleviate experimental OA lesions after intra-articular administration (55). In addition, intervention in IL-10/IL-10Ralpha-related glycolytic responses in synovial macrophages can improve cartilage degeneration in mouse OA (56). These studies use different intervention pathways, but they all show that established inflammatory and metabolic states of macrophages can still be changed. They do not yet prove that metabolic abnormalities initiate persistent synovitis, nor do they answer whether these states can recover spontaneously after injury stimuli are removed. For synovial immune control failure, the significance of metabolic change may lie more in influencing how long macrophages remain in a functional state than in constituting a fixed metabolic pattern shared by all OA.

Disease-associated changes in FLS are also not limited to increased inflammatory mediators. They involve coordinated changes in metabolism, proliferation, senescence, and stromal remodeling. In primary OA-FLS, abnormal PDK3 expression is associated with enhanced glycolysis and active cell proliferation. After PDK inhibition, cellular metabolism shifts toward oxidative phosphorylation, with reduced proliferation and inflammatory mediator release (57). In diabetes-related experimental OA, intervention in YAP1-regulated FLS glycolysis reduces synovial macrophage infiltration and improves joint pathology (58). In addition to metabolic changes, ROS/TGF-beta-related signaling can promote SOX4 accumulation in OA-FLS and enhance senescence-associated phenotypes (59). These findings show that metabolic, inflammatory, and tissue-remodeling functions in FLS are not isolated from one another, and that disease-associated FLS states are partly plastic. However, existing studies mainly observe changes after pharmacological or genetic intervention, and they do not determine whether these states can naturally exit after injury input weakens.

Interactions between macrophages and FLS may further prolong retention of these states. Extracellular vesicles released by inflammatory FLS can be taken up by macrophages and enhance their glycolysis and inflammatory responses. Intra-articular administration of these vesicles can also aggravate synovitis and OA pathology in animals (60). Single-cell analysis of human OA synovium has identified oncostatin-related communication between monocyte-derived macrophages and FLS, and functional experiments show that this signal can alter inflammatory and matrix-remodeling programs in FLS (53). These studies provide direct evidence of bidirectional regulation between the two cell types. They have not shown that this connection can independently maintain inflammation in the absence of ongoing tissue injury. A more reasonable interpretation is that external injury input first changes local cellular states, and repeated communication among cells then makes these changes harder to resolve in time.

Adaptive immune responses in OA synovium vary markedly by patient and disease stage. CD4+ T-cell infiltration can be detected in synovial fluid and synovium from patients with early knee OA. Joint-derived T cells show a clearer Th1-related phenotype, including increased expression of CXCR3, CCR5, and IFN-gamma. The proportion of Treg cells in the synovium is also higher than in synovial fluid and peripheral blood (61). In patients with late-stage knee OA, the degree of synovial infiltration by different helper T-cell subsets correlates with pain, functional impairment, and disease severity (62). These findings indicate that T cells participate in synovial responses in some patients with OA. However, existing clinical studies mainly describe cell infiltration and phenotypic associations, and they do not determine whether T cells directly maintain abnormal macrophage and FLS states.

Treg cells may represent a counter-regulatory component in synovial inflammation. Paired analyses show that the proportion of Treg cells in OA joint-derived samples is higher than in peripheral blood, and that a lower proportion of synovial Treg cells is associated with worse pain and functional impairment (63). Reduced IL-10 secretion by Treg cells in patients with OA is also associated with decreased Tim-3 expression (64). These observations suggest that pro-inflammatory T-cell responses and insufficient immune regulation may coexist, but their influence on the duration of synovial inflammation requires further validation. RA studies have shown in detail that FLS can regulate T-cell activation (65). Whether this cellular interaction exists in OA in the same way still lacks direct functional evidence.

B-cell responses are likewise not shared by all patients with OA. Clonal expansion of B cells can be seen in some OA synovia. Samples with more prominent B-cell infiltration often show more severe synovitis, and a small number of cases can show plasma cells and lymphoid follicle-like structures (66, 67). Recent studies also show that B cells from patients with OA have altered expansion and plasma-cell differentiation capacity, while retaining antibody-producing ability (68). These changes are not present in all patients with OA, and there is no consensus on their antigen specificity or pathological significance. Compared with the clearer lymphocyte clonal expansion and organized responses in RA synovium (69), adaptive immunity in OA more likely represents a specific inflammatory endotype rather than a universal initiating mechanism.

In summary, macrophages, FLS, and adaptive immune cells in OA synovium can all show disease-associated states, but the combinations of these cells differ among patients. Existing intervention studies indicate that some cellular states are plastic. They have not yet shown that these states can reset spontaneously after stimuli weaken, nor have they identified which cellular relationships are retained over the course of disease. Synovial immune control failure focuses precisely on whether disease-associated cellular states cannot exit promptly after the original injury weakens, and whether local cellular communication further prolongs this process.

### 3.4 The synovial inflammatory niche: an organized state of persistent inflammation

When damage-related signals persist and clearance, inflammatory resolution, and cellular-state resetting are not completed in time, abnormal responses in the synovium may no longer be limited to individual cells. Macrophages, FLS, and some lymphocytes are repeatedly exposed to similar local signals, allowing programs related to inflammation, chemotaxis, and tissue remodeling to persist in the same region. At this stage, synovial inflammation no longer reflects only an immediate response to a particular tissue injury. It reflects a tissue state that is difficult to restore. This article uses the term synovial inflammatory niche to describe this state. Its core is not an increase in one cell type or persistent activation of one inflammatory pathway. It is the local retention of disease-associated cellular states and their relationships. Injury input can promote formation of this state, while persistent cellular communication and changes in the tissue environment may prolong its duration. Therefore, inflammatory intensity may not always remain synchronized with the current degree of tissue injury. Some patients may show recurrent or long-standing synovitis even without continuously increasing acute injury.

Current OA studies have identified disease-associated macrophage and FLS states and have provided evidence for functional communication between the two cell types. These findings suggest that persistent synovitis is not maintained by a single cell type alone. However, they do not yet confirm that the relevant cellular states form stable and long-lasting spatial structures. Most human synovial samples come from patients undergoing late-stage joint replacement, and cross-sectional studies cannot determine whether these changes are gradually retained during disease progression or are accompanying features of severe tissue damage. The synovial inflammatory niche therefore remains an integrative explanation for a persistent tissue state. Future studies need to determine whether the relevant cellular states can coexist persistently in specific regions and fail to reset in time after injury input weakens. The inflammatory niche is not a closed system that operates apart from other joint tissues. It is a tissue-level manifestation of whole-joint pathological feedback within the synovium.

## 4 Crosstalk between the synovium and other joint tissues

The injury signals received by the synovium do not arise only within the synovium. Cartilage, subchondral bone, and the meniscus each have their own processes of mechanical stress, cell injury, and tissue remodeling. These changes can occur before obvious synovitis, or they can progress together with synovial lesions. Matrix fragments, soluble mediators, and mechanical changes produced by these tissues continuously alter the local joint environment. Once synovial responses persist, they can in turn affect cartilage metabolism, bone remodeling, and neurovascular changes. Synovial immune control failure should be understood within this cross-tissue context. The synovium can serve as an interface where pathological signals converge and are re-exported, but it does not have to be the common first lesion in all patients with OA.

### 4.1 Cartilage injury and synovial responses

Articular cartilage is exposed to long-term compression, shear, and repeated loading. Abnormal load distribution, age-related cellular changes, and metabolic stress can disrupt the balance between matrix synthesis and degradation in chondrocytes. As the proteoglycan and collagen networks are damaged, cartilage matrix fragments, chondrocyte secretions, and extracellular vesicles enter synovial fluid, placing the synovial lining in continuous contact with cartilage-derived injury input. Studies of molecular communication among cartilage, synovium, meniscus, and subchondral bone have shown extensive paracrine and matrix-signal exchange among tissues in OA joints (70). Cartilage degeneration can therefore actively alter the environment of the synovium, rather than being only a tissue outcome after synovial inflammation.

Cartilage-synovium co-culture experiments provide more direct evidence for functional links between the two tissues. When bovine cartilage is co-cultured with synovium or fibrous joint capsule, aggrecanase and matrix metalloproteinase activity increase in cartilage tissue, with increased proteoglycan degradation (71). In human cartilage-synovium explant models, co-culture of the two tissues also induces interconnected changes in inflammatory mediators and matrix metabolism (72). Such models preserve part of tissue structure and cellular composition and are closer to the intra-articular environment than single-cell stimulation. They have therefore been used to evaluate cross-tissue responses and candidate OA therapies (73). However, co-culture systems usually lack physiological loading, intact circulation, and long-term disease evolution, so they cannot determine which tissue changes first in human OA.

The effect of the synovium on cartilage can be mediated by soluble factors and extracellular vesicles. Exosomes released by IL-1beta-stimulated synovial fibroblasts can induce inflammatory and matrix-degradation-related changes in chondrocytes (74). Small extracellular vesicles derived from OA-FLS have miRNA compositions different from those of non-OA cells, and after uptake by chondrocytes they can enhance catabolic responses (75). PCGEM1 carried by FLS exosomes can aggravate IL-1beta-induced chondrocyte apoptosis and matrix degradation through a miR-142-5p/RUNX2-related mechanism (76). Vesicles from synovial fibroblasts carrying miR-21-5p can also enhance OA-related inflammatory responses (77). These experiments confirm that synovium-derived vesicles are biologically active. However, most studies use *in vitro* stimulation or high-dose intra-articular administration, and the actual abundance and relative contribution of vesicles from different sources in human OA synovial fluid remain unclear.

In addition to soluble and vesicular signals, molecules related to cell connections may affect responses between joint tissues. Modulation of connexin 43 can alter inflammatory and matrix metabolic phenotypes in human chondrocytes, osteoblasts, and cartilage explants (78). This study does not directly prove that synovial cells and chondrocytes exchange information stably through connexin 43 *in vivo*. It does suggest that intercellular connections and small-molecule exchange may participate in joint tissue responses, and this role still needs confirmation in spatial models containing both synovium and cartilage.

Feedback from cartilage injury to the synovium does not depend only on the quantity of fragments, but also on whether the fragments are biologically active. Fibronectin fragments can induce expression of multiple matrix metalloproteinases in human chondrocytes through MyD88-dependent TLR2 signaling (79). Peptides derived from proteoglycan aggrecan can also enhance expression of catabolic mediators in chondrocytes through TLR2-related pathways (80). These studies mainly examine responses in chondrocytes themselves and do not directly prove that the same fragments necessarily activate OA synovial macrophages or FLS in the same manner. However, they show that cartilage matrix degradation products are not inert debris and may continue to participate in intra-articular signaling. Whether their entry into synovial fluid prolongs synovial inflammation needs to be assessed by integrating synovial-fluid concentrations, synovial clearance capacity, and direct cell-stimulation experiments.

Cross-tissue cellular communication also differs by disease context and anatomical site. A single-cell study of temporomandibular joint OA found that different types of occlusal disorder can generate different immune cell-chondrocyte communication patterns (81). This finding cannot be directly generalized to knee OA, but it indicates that the type of mechanical injury may change communication between cartilage and immune cells. Similar contextual differences may exist in knee OA. For example, synovial responses caused by acute trauma, long-term abnormal loading, and age-related degeneration may not be the same.

The cartilage-synovium relationship should not be described as a fixed one-way cascade. In patients with mild synovitis, abnormal mechanical loading and intrinsic chondrocyte changes may remain dominant for a long time. In patients with more obvious synovitis, synovium-derived mediators and extracellular vesicles can increase catabolic pressure on cartilage. Newly generated matrix fragments can then re-enter synovial fluid and further increase the clearance and regulatory burden of the synovium. In this loop, the role of synovial immune control failure is to make cartilage-derived injury inputs harder to process in time and to prolong the duration of inflammatory and catabolic signals sent from the synovium to cartilage. It does not reduce cartilage degeneration to a downstream result of synovial pathology.

### 4.2 Subchondral bone remodeling and synovial inflammation

Subchondral bone does not passively become sclerotic only after severe cartilage wear. Osteocyte sensing of abnormal mechanical loading, focal osteoclastic activity, changes in the bone marrow environment, and imbalance in osteoblast-osteoclast coupling can all appear early in OA. Changes in the number, morphology, and spatial distribution of the microchannel network have already been observed in early OA subchondral bone (82). Mouse knee OA models also show that RANKL-mediated subchondral bone resorption can appear at an early stage and precede later cartilage degeneration and uncoupled bone remodeling (83). This evidence indicates that subchondral bone has a relatively independent pathological process and should not be regarded simply as a secondary structural change after synovitis or cartilage wear.

Under normal conditions, hyaline cartilage, the calcified cartilage layer, the tidemark, and the subchondral bone plate form a tissue separation between the joint cavity and the bone marrow environment. As OA progresses, deep cartilage fissures, disorganization of the calcified cartilage layer, and microdamage to the subchondral bone plate weaken this separation. Vascular invasion after cartilage injury is thought to be related to local matrix changes, angiogenic signals, and remodeling of the osteochondral interface (84). Elevated PDGF-BB in subchondral bone can promote angiogenesis and is associated with experimental OA progression (85). Increased vascularization can also be observed in the subchondral region of osteoarthritic femoral heads in older adults (86). These changes increase the possibility of material exchange among bone marrow, the osteochondral junction, and the joint cavity. However, most human data currently come from end-stage tissue observations, and the specific transmission functions of microchannels and new vessels in early disease remain uncertain.

Subchondral bone remodeling involves multiple interconnected processes. After RANKL binds RANK, it can promote osteoclast differentiation and fusion through TRAF6, NF-kappaB, MAPK, c-Fos, and NFATc1 signaling (87–89). When osteoclastic resorption increases in early OA, TGF-beta stored in the bone matrix can be released and can affect recruitment of bone marrow mesenchymal progenitors and abnormal bone formation. Inhibition of TGF-beta signaling in subchondral bone mesenchymal stem cells can reduce experimental OA lesions (90). Disruption of the hypoxic state of subchondral bone is also related to abnormal angiogenesis and bone remodeling, and maintenance of the local hypoxic environment can slow OA progression in animal models (91). Endothelial PDGF-BB/PDGFR-beta signaling can promote abnormal subchondral bone formation in an angiogenesis-dependent manner (92). These studies reflect local remodeling jointly involving bone cells, vascular endothelial cells, and mesenchymal cells. This process cannot be explained entirely as spread of synovial inflammation into deep tissues.

Synovial inflammation can add additional influence to this local program. CCL2/CCR2 signaling participates in monocyte recruitment within OA joints and is associated with synovitis, inflammatory mediator expression, and cartilage destruction (93). This pathway may increase the local supply of myeloid cells in the joint, but current studies have not shown that monocytes recruited into the synovium directly migrate to subchondral bone and become osteoclasts. PGE2/EP4 signaling has more direct evidence in subchondral bone. PGE2 acting on EP4 in subchondral bone osteoclasts can promote osteoclastic activity, PDGF-BB expression, and subsequent vascular and bone remodeling changes, and blockade of this pathway can alleviate experimental OA (94). Therefore, PGE2 produced by the synovium or other joint tissues may influence subchondral bone remodeling, but its main tissue source still needs to be distinguished.

Studies of RANKL blockade further show a link between synovitis and bone remodeling. In experimental knee OA, denosumab not only alters RANK-related bone remodeling but also reduces synovial inflammation and disease progression through RANK/TRAF6/FSTL1-related signaling (95). This result suggests that bone resorption and synovitis may share signaling nodes. It does not imply that subchondral bone changes must be driven first by the synovium. When a drug acts on multiple tissues, improvement in the synovium may result from reduced bone-derived signals, or from inhibition of RANK-related responses in immune cells.

Subchondral bone lesions can also affect pain and the signaling environment in which the synovium is located. Subchondral bone osteoclasts can promote sensory nerve ingrowth and participate in OA pain formation (96). Microinjury at the osteochondral junction, abnormal bone turnover, vascular dilation, and increased nerve fibers make this region a site where structural changes and pain signals converge (97). Whether related signals reach the synovium directly still lacks continuous tracing evidence. However, bone marrow lesions and neurovascular remodeling can alter inflammatory and sensory inputs across the whole joint and thereby affect synovial responses and clinical symptoms.

The subchondral bone-synovium connection is a bidirectional process that changes with disease stage. At an early stage, abnormal loading, osteocyte responses, and focal osteoclastic activity may dominate. As the osteochondral interface gradually changes, exchange between synovium-derived mediators and bone-derived signals becomes easier. Synovial inflammation can increase myeloid cell recruitment and PGE2- and RANKL-related responses. Subchondral bone microinjury, abnormal vascularization, and nerve ingrowth can provide new structural, metabolic, and pain-related inputs. Which side is dominant in a given patient needs to be judged together with synovitis, bone marrow lesions, bone turnover, and pain phenotype.

### 4.3 Whole-joint injury inputs and outcomes of synovial responses

**Figure 3:**
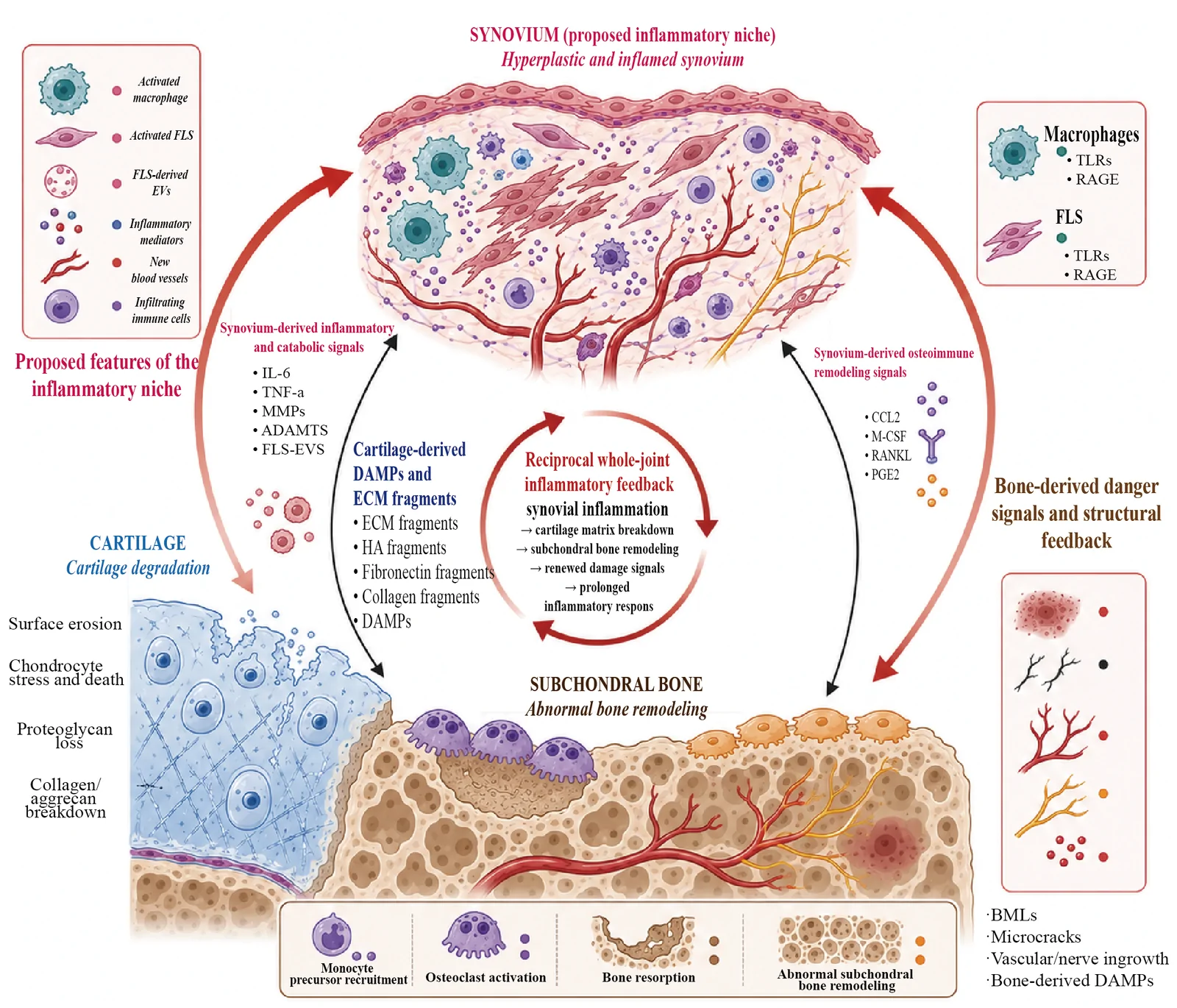
Reciprocal interactions among the synovium, cartilage, and subchondral bone in OA. Cartilage degeneration, meniscal injury, altered mechanical loading, and subchondral bone remodeling can generate matrix fragments, extracellular vesicles, soluble mediators, and other damage-related signals that reach the synovium. These inputs may alter macrophage and FLS states and increase inflammatory, chemotactic, and matrix-remodeling activity. In turn, synovium-derived cytokines, proteases, lipid mediators, and extracellular vesicles may increase chondrocyte stress and matrix catabolism and may influence myeloid-cell recruitment, osteoclast activity, angiogenesis, and subchondral bone remodeling. Bone marrow lesions, microdamage at the osteochondral junction, abnormal vascularization, and sensory nerve ingrowth may provide additional structural, metabolic, and pain-related inputs to the joint environment. These reciprocal interactions may prolong synovitis and whole-joint tissue remodeling, particularly when continued damage input coexists with impaired synovial recovery. The proposed inflammatory niche represents the local retention of disease-associated synovial cell states and their interactions; it is not intended to establish the synovium as the common initiating tissue in all patients with osteoarthritis. The schematic is based on representative studies of cartilage–synovium communication, extracellular-vesicle signaling, matrix-derived danger signals, subchondral bone remodeling, angiogenesis, osteoclast activity, and osteoarthritis pain.

Cartilage, meniscus, subchondral bone, and the fat pad can all provide damage-related signals to the synovium. However, the degree and duration of synovitis cannot be inferred directly from structural damage in any single tissue. Similar degrees of cartilage wear or bone marrow lesions can be accompanied by different degrees of effusion and synovial thickening. Conversely, synovitis in some patients can persist even when structural damage shows little change. This difference is related to the source, intensity, and duration of injury signals, and also to whether the synovium can complete debris clearance, inflammatory resolution, and cellular-state resetting. Therefore, whole-joint injury and persistent synovitis do not have a fixed linear relationship. When mechanical abnormalities and tissue destruction continuously generate new signals, synovitis mainly reflects persistent external input. When injury burden is relatively stable but the local response still cannot end, impaired recovery of the synovium itself may play a larger role. The two processes can coexist in the same patient and can change in relative weight with disease stage. Synovial immune control failure is not the unified starting point of OA. It is a possible mechanism that explains why multi-tissue injury is transformed into long-term inflammation and repeated cross-tissue feedback in some patients (**Figure 3**).

## 5 Synovial recovery capacity: model testing and disease stratification

The most direct manifestation of synovial immune control failure should not be a higher inflammatory level at one time point. It should be a delay in the process by which the synovium returns to a low-inflammatory state after injury input weakens. If synovitis is only an immediate response to cartilage wear, meniscal injury, or subchondral bone abnormality, its changes should generally fluctuate with worsening or improvement of these lesions. Existing longitudinal studies show that cartilage and subchondral bone changes can predict changes in effusion-synovitis, while persistent effusion-synovitis is associated with later cartilage damage and whole-joint structural degeneration. This bidirectional but asynchronous relationship provides a clinical basis for distinguishing persistent injury input from impaired synovial recovery.

This judgment first requires longitudinal studies, rather than relying only on joint replacement samples or single imaging time points. Continuous follow-up of patients with similar degrees of cartilage injury, bone marrow lesions, and meniscal abnormalities can compare whether structural lesions and synovitis change synchronously. Previous studies have observed relationships between effusion-synovitis and joint structural changes over 24-month, 18-month, and 4-year periods (98–100). If the duration of synovitis still differs markedly under similar structural injury backgrounds, and this difference is related to later pain or structural progression, synovial clearance and resolution functions should be examined further. If synovitis always follows changes in tissue injury and resolves promptly after injury is reduced, immune control failure has less explanatory value for those patients.

Ex vivo studies can further distinguish enhanced inflammatory responses from impaired recovery. Synovial tissues, macrophages, or FLS from different patients can be exposed to the same short stimulus and then observed continuously after stimulus withdrawal. Human OA synovial explants can preserve tissue structure, cellular composition, and inflammatory response features. Multi-tissue microphysiological culture systems can also observe dynamic links among mechanical injury, synovial inflammation, and matrix degradation (101–103). On this basis, the key assessment should not remain at the inflammatory peak after stimulation. It should examine whether inflammatory output declines, whether clearance function recovers, and whether disease-associated cellular states exit. If samples from patients with persistent synovitis recover more slowly under the same conditions, the difference may be related to the state of the synovium itself. If all groups recover rapidly after stimulus withdrawal, persistent inflammation is more likely to depend on continuous external injury input.

Intervention studies should also focus on the duration of inflammation, not only the decline of a single marker. In experimental OA, pro-resolving lipid mediators can regulate synovial macrophage states and reduce synovitis (104). Activation of Gas6/Axl signaling can reduce inflammatory output from human OA-FLS and synovial explants (105). Stabilized anti-inflammatory macrophages can also maintain relatively stable regulatory and repair functions in the OA microenvironment (106). These studies have not directly proved that synovial immune control has been restored, but they provide feasible intervention directions for functional testing. If an intervention only lowers the inflammatory peak but does not shorten the duration of synovitis or change the retention of abnormal cellular states, it is insufficient to prove recovery of immune control. Conversely, if an intervention accelerates synovitis resolution without obvious change in the initial tissue injury, this suggests that intrinsic regulatory capacity of the synovium contributes to inflammation maintenance.

This approach can also provide a new basis for OA stratification. Previous synovial fluid and synovial studies have identified OA patient subgroups with different myeloid-cell, metabolic, and inflammatory features, including synovial subgroups with different degrees of macrophage proliferation and effusion-type knee OA patients with different local inflammatory and metabolic profiles (107, 108). In addition, studies based on clinical manifestations, molecular features, and biomarkers suggest that OA is not a single disease process, but contains different molecular endotypes and disease trajectories (109, 110). However, existing classifications mainly reflect inflammatory or structural states at a single time point. They do not explain why some patients show persistent synovitis despite similar degrees of injury. On this basis, patient differences can be further assessed from two directions. One is the continuous injury input provided by cartilage, meniscus, and subchondral bone. The other is the ability of the synovium to clear, restrict, and terminate these inputs. When neither injury input nor synovial recovery capacity is obviously abnormal, synovitis may mainly change with tissue injury. When injury input is limited but recovery capacity declines, synovitis may show stronger persistence. When both abnormalities coexist, a more stable whole-joint feedback loop may form.

Corresponding identification markers should also be built around these two dimensions. Repeated MRI or ultrasound can be used to observe whether synovitis changes synchronously with cartilage, meniscal, and subchondral bone changes (98–100). Macrophage- and neutrophil-related molecules in synovial fluid, soluble macrophage markers, and synovial cellular composition can reflect biological heterogeneity of local inflammation (111). At present, these markers mainly reflect the degree of inflammation or cellular composition, and they cannot by themselves prove impaired recovery capacity. The value of macrophage and FLS states ultimately depends on whether they can be linked to the persistence trajectory of synovitis and whether these states can recover after stimuli weaken. At this stage, this framework is used to propose a testable approach to patient stratification. It does not constitute a clinical diagnostic standard.

## 6 Therapeutic strategies

Anti-inflammatory treatment for OA has not produced consistent clinical results. Traditional antirheumatic drugs have limited overall efficacy in unstratified OA populations (112). However, in patients with hand OA and MRI-detected synovitis, methotrexate can reduce pain, suggesting that some patients with clear inflammatory features may still benefit from immune modulation (113). Denosumab showed some structural improvement in a randomized trial of erosive hand OA, but its effect may involve both bone remodeling and inflammatory responses and cannot be simply attributed to synovial suppression (114). Differences among studies indicate that synovitis is not a common and stable therapeutic target in all patients with OA. Patient selection may be more important than simply increasing anti-inflammatory intensity.

Treatment selection also needs to distinguish the main source of persistent synovitis. When mechanical instability, meniscal injury, cartilage degeneration, or subchondral bone abnormalities continue to progress, the synovium continuously receives new injury input. In this setting, controlling inflammation alone is unlikely to produce durable effects. If structural status is relatively stable but synovitis continues to recur, insufficient clearance, delayed inflammatory resolution, or failure of cellular-state resetting may have greater therapeutic relevance. The two processes can also coexist. Structural intervention and synovial therapy are therefore not mutually exclusive options.

Intervention in synovial immune activity should also not aim at global suppression. After mouse joint injury, intra-articular depletion of macrophages with clodronate liposomes does not reduce synovitis and instead aggravates acute inflammation (34). This finding is consistent with the fact that macrophages also perform debris clearance and inflammation-restricting functions. By contrast, targeting STAT6-related macrophage states can improve pain in experimental OA (115). Resolvin D1 nanoliposomes can regulate synovial macrophage responses and reduce joint lesions (116). Inhibition of PIM-1 can also reduce macrophage NLRP3 inflammasome activation and alleviate synovitis and structural damage in DMM mice (47). A unified macrophage target cannot yet be defined, but the different outcomes of broad depletion and functional modulation suggest that therapy should preserve tissue-maintenance functions.

FLS therapy should likewise avoid nonselective suppression. In primary OA-FLS, inhibition of abnormally elevated pyruvate dehydrogenase kinases can reduce glycolysis, cell proliferation, and inflammatory mediator release (57). Small extracellular vesicles derived from OA-FLS can enhance catabolic responses in chondrocytes (75), and experimental interventions targeting pathological FLS-derived vesicles or particles have also shown some protective effects (117). These studies provide a basis for limiting pathological FLS states and abnormal secretory communication. They do not support long-term suppression of all FLS. Lining FLS still participate in maintaining lubricin, hyaluronic acid, and the surface matrix. The therapeutic target should be persistent pathological function, not the cell population itself.

Some interventions may act on both the synovium and subchondral bone. In experimental OA, RANKL blockade reduces synovitis while improving bone remodeling (95). PGE2/EP4 signaling participates in subchondral bone osteoclastic activity, angiogenesis, and subsequent bone structural changes (94). These findings indicate that bone and synovium have pathological links that can be targeted together. However, when a drug acts on multiple tissues, it is also harder to determine the main source of efficacy. Therefore, in patients with prominent subchondral bone marrow lesions or abnormal bone turnover, combined evaluation of bone remodeling and synovial changes is more meaningful than observing synovial inflammatory markers alone.

Evaluation of efficacy should also shift from single inflammatory measurements to the time course of inflammation. Longitudinal studies have found bidirectional links between effusion-synovitis and cartilage and subchondral bone changes (98). Changes in synovitis are also associated with later progression of cartilage damage (99). Persistent synovitis can accompany more obvious long-term whole-joint structural degeneration (100). Therefore, in addition to assessing pain, inflammatory mediators, and structural changes, future clinical studies should record the time needed for synovitis resolution, recurrence after treatment withdrawal, and whether abnormal cellular states recover. Only when an intervention in properly stratified patients shortens the duration of inflammation is there reason to think that it improves synovial immune control rather than temporarily suppressing inflammatory output.

## 7 Summary and outlook

This article does not aim to show again that the synovium participates in OA. It aims to explain why synovitis fails to end in some patients. In homeostasis, synovial macrophages, FLS, and the vascular-interstitial interface jointly process intra-articular debris, restrict inflammatory spread, and promote local tissue recovery after injury weakens. When cartilage, meniscus, and subchondral bone continue to generate injury signals, synovitis can worsen accordingly. In some patients, however, persistence of inflammation may not be fully explained by new injury input. When debris clearance, inflammatory resolution, and cellular-state resetting become gradually impaired, the tissue state induced by earlier injury may be retained. Abnormal responses of macrophages and FLS, together with persistent communication between the synovium and other joint tissues, further prolong local inflammation. The synovial inflammatory niche described here summarizes this persistent tissue state and remains a pathological explanation that requires validation.

Synovial immune control failure does not mean that the synovium is the common starting point of all OA. Abnormal mechanical loading, intrinsic chondrocyte changes, meniscal injury, and subchondral bone remodeling can independently or jointly promote disease progression. In some patients, synovitis remains limited even when structural damage is obvious. Even among patients with persistent synovitis, the tissue basis may differ. Some cases may be driven mainly by continuous generation of new injury signals from joint tissues, whereas others may involve delayed synovial recovery. The two processes can also coexist. A single imaging finding of synovial thickening, effusion, or increased inflammatory mediators cannot distinguish these situations. Compared with how strong inflammation is at one time point, whether the synovium can recover promptly after structural injury weakens may be closer to the pathological feature emphasized by this model.

Whether this hypothesis is valid depends on temporal and functional evidence. Longitudinal studies need to determine whether synovitis always changes synchronously with cartilage, meniscal, and subchondral bone lesions, and whether local inflammation can continue after structural injury becomes relatively stable. Ex vivo tissue and cell studies should observe inflammatory output, debris clearance, and recovery of cellular states after stimulus withdrawal, rather than only comparing peak responses after stimulation. Multi-time-point spatial analysis can further determine whether abnormal relationships among macrophages, FLS, and adaptive immune cells remain after injury weakens. If synovitis always follows tissue injury and recovers rapidly after stimulus withdrawal, synovial immune control failure has limited explanatory value for those patients. If delayed recovery can be repeatedly observed in patient samples and experimental models and can be reversed by interventions that promote clearance or inflammatory resolution, the model will gain substantive support.

For clinical practice, the value of this framework is not to establish another fixed OA classification system, but to add a dynamic dimension to patient heterogeneity. Structural imaging can be used to judge whether injury input is still ongoing. Repeated MRI or ultrasound can record the trajectory of synovitis. Synovial fluid and synovial cell analyses can help evaluate clearance, resolution, and cellular-state resetting. Combining the source of injury with synovial recovery capacity may help explain the inconsistent results of some anti-inflammatory trials and identify patients more likely to benefit from synovial intervention. At present, this idea still cannot be used as a clinical diagnostic or treatment-selection standard. Its scope of application, evaluation markers, and predictive value need to be determined in longitudinal cohorts and stratified intervention studies.

## Data Availability

The original contributions presented in the study are included in the article/supplementary material. Further inquiries can be directed to the corresponding authors.
